# Quantitative Serum Proteomic Analysis of Essential Hypertension Using iTRAQ Technique

**DOI:** 10.1155/2017/6761549

**Published:** 2017-10-22

**Authors:** Jing-Wen Xu, Yun-Lun Li, Shi-Jun Zhang, Wen-Qing Yang, Wen-Ting Nie, Hai-Qiang Jiang

**Affiliations:** ^1^Shandong University of Traditional Chinese Medicine, 4655 Daxue Road, Changqing District, Jinan, Shandong Province, China; ^2^Affiliated Hospital of Shandong University of Traditional Chinese Medicine, 16369 Jingshi Road, Lixia District, Jinan, Shandong Province, China; ^3^Affiliated Hospital of Shandong Academy of Medical Sciences, 38 Shadowless Hill Road, Tianqiao District, Jinan, Shandong Province, China

## Abstract

Essential hypertension (EH) is a risk factor for some severe diseases. This study aimed to screen out serum special proteins and seek interaction between them, which would provide new therapeutic targets and elucidate the comprehensive pathophysiological mechanism for EH. Patients with EH (Group A, *n* = 47) and healthy controls (HC) (Group B, *n* = 47) were recruited in this study. Serums from the two groups were analyzed with isobaric tags for relative and absolute quantitation coupled two-dimensional liquid chromatography followed by electrospray ionization-tandem mass spectrometry technique, while the candidate special proteins were verified with ELISA and western blot. A total of 404 proteins were identified, of which 30 proteins were upregulated (>1.2-fold, *p* < 0.05) and 81 proteins were downregulated (<0.833-fold, *p* < 0.05) compared with HC group. With GO, KEGG analysis, and literature retrieval, 4 proteins, cathepsin G, transforming growth factor beta-1, hyaluronidase-1, and kininogen-1, were found jointly involved in the renin-angiotensin-aldosterone system and kallikrein-kinin system. The profiles of these 4 candidate proteins were confirmed with ELISA and western blot. The concentration variation of these 4 proteins could better predict the occurrence and illustrate the pathophysiological mechanism of EH. And their discovery may help pave the way for exploring new therapies of EH.

## 1. Introduction

Essential hypertension (EH) is an age-dependent disease and is defined as pathological high blood pressure (BP) [1]. The risk of cerebral, cardiac, and renal events would be increased [2, 3]. In China, there has been a strong upward tendency in EH incidence with age. Until 2010, about 20 percent of people in China have suffered from high BP [4]. However, the comprehensive pathogenic mechanism of EH is still unclear, which resulted in unsatisfactory prevention and treatment effects at the present stage. Therefore, further studies in depth should be performed on the internal pathomechanism of EH, which would be clinically valuable in early prevention and control of EH.

So far, lots of scholars have studied the pathogenic mechanism of EH from genetics and metabonomics. However, the middle link between genetics and metabonomics was always ignored, whose level and activity variation would directly have effects on the EH process. The physical functions would be performed through protein interaction. Thus, manifestations would be merely associated with protein interaction, which also indicated that protein was the direct executor of vital activities [5]. It is considered that proteomics variation would be the middle link between genetics and metabonomics for pathophysiological reactions. Meanwhile, as proteins keep varied in all stages of life activities, they could dynamically respond to genetic manipulation, endogenous variation, and exogenous stimuli [6]. It is assumed that disordered proteins may play a key role in BP elevation and the abnormal BP must be reflected by a special protein manifestation. It would be involved in an integral physiological function.

Blood travels through the human body and contains various products of physiological and pathological processes. Most abundant secreted factors can be observed in the blood and thus it was recognized as a highly believable sample for disease-related biomarkers [7]. After separating blood cells and fibrin, the remaining supernatant was serum. Serum is also an ideal sample for biological analysis, which retains rich bioinformation. Serum proteomics has gained considerable interest during disease biomarker exploration [8]. In this study, it was also chosen as the research sample.

In this study, serum proteins were identified and quantified in both EH individuals and healthy controls with iTRAQ labeling coupled with 2D LC-MS/MS technique. First, the candidate differentially expressed proteins were filtered in previous discovery section. Second, 4 special candidates were selected out according to GO annotation, KEGG pathway, and literature analysis in further discovery section. Third, the 4 candidates were validated with ELISA and western blot for confirming their variation tendency in discovery section.

## 2. Methods and Materials

### 2.1. Study Design and Subjects

This study design was reviewed and approved by the Ethics Committee of the Affiliated Hospital of Shandong University of Traditional Chinese Medicine (Jinan, China). The study proposal was approved by the institutional review board. All patients signed written informed consent. Serum samples of both healthy individuals and EH patients were obtained from the Outpatient Department of Cardiology of the Affiliated Hospital of Shandong University of Traditional Chinese Medicine (Shandong, China) and Jinan Hospital of Traditional Chinese Medicine (Shandong, China).

All EH patients were defined and screened according to the criteria of Hypertension Prevention and Cure Guideline of China in 2010. It included 47 patients (age range: 43–62 years old) meeting the BP criteria (140 mm Hg ≤ SBP ≤ 179 mm Hg or 90 mm Hg ≤ DBP ≤ 109 mm Hg) after a washout period of two weeks, who were regarded as Group A, while another 47 healthy volunteers meeting the age requirement were regarded as Group B.

*Exclusion Criteria*. Individuals that fell outside the age range and BP range; patients diagnosed with secondary hypertension; pregnant women, breastfeeding women, and women planning to be pregnant; patients with allergic conditions; and patients with diabetes mellitus, uncontrolled hypertension, heart failure, mental disorder, renal dysfunction, or liver disease were all excluded.

**Figure 1:**
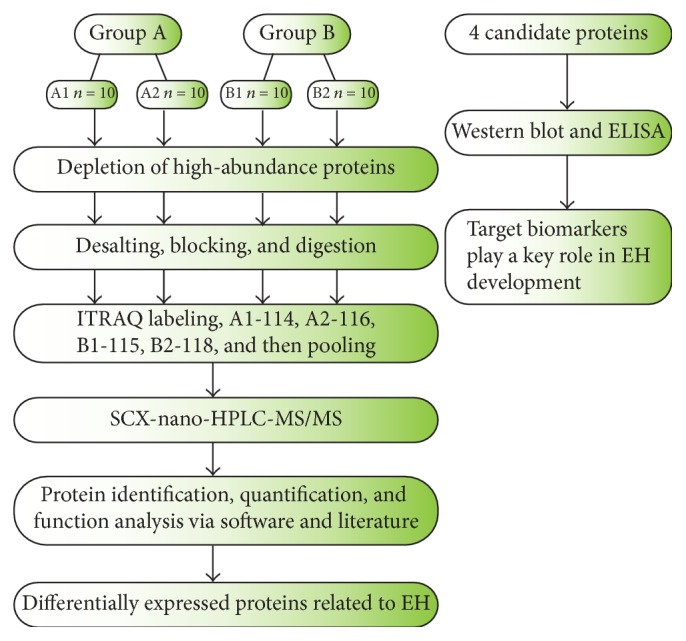
Overview of the workflow for discovering and verifying candidate special proteins (Group A: HC; Group B: EH). The left section was the discovery phase; the individuals in each group were randomly divided into two subgroups for biological repeat (A1, *n* = 10; A2, *n* = 10; B1, *n* = 10; B2, *n* = 10). Then, the right section was the verification phase; a total of 54 individuals were involved, including 27 HC and 27 EH.

 All the enrolled individuals underwent a standardized clinical examination including body mass index (BMI), BP, AngII (angiotensin II), and ALD (aldosterone) (Table 1). The workflow of this study was described (Figure 1).

### 2.2. Sample Preparation

20 individuals in each group were randomly selected for proteomics analysis in discovery section. Morning fast blood (5 mL) was collected with serum separation tubes. After clotting for 30 min at room temperature, samples were centrifuged for 10 min at 4000 rpm for serum separation. Then, the supernatant was centrifuged for 10 min at 10,000 rpm and lipid in upper lay was removed. At last, the supernatant was collected into EP tubes and stored at −80°C till use. Hemolysis should be avoided in sample preparation. Serum samples were pooled for the next step (10 samples of each group were pooled together; Groups A1, A2, B1, and B2 were formed). For minimizing the interference from complex samples, the highly abundant proteins were depleted with ProteoMiner™ Kits (Bio-Rad Laboratories, Hercules, CA, USA).

### 2.3. Trypsin Digestion, ITRAQ Labeling, and SCX Fractionation

Samples were diluted in lysis buffer (7 mol/L urea, 2 mol/L thiourea, 4% CHAPS, and 40 mmol/L Tris-HCl, pH 8.5) and reduced with 10 mmol/L DTT (final concentration after mixing) at 56°C for 1 h. The samples were then alkylated with 55 mmol/L IAM (final concentration) in a dark room for 1 h. After reduction and alkylation, protein mixture was precipitated by adding 4x volume of chilled acetone and staying still at −20°C overnight. After centrifuging at 30000*g* at 4°C, the protein pellet was collected. Then, the pellet was dissolved in 0.5 M TEAB (Applied Biosystems, Milan, Italy) and sonicated in ice. After centrifuging at 30,000*g* at 4°C, an aliquot of the supernatant was taken for determining protein concentration with Bradford assay (Solarbio, Beijing, China) [9]. Proteins (100 *μ*g) from each sample were typically digested and labeled with 8-plex iTRAQ reagents (Applied Biosystems, USA).

The labeled samples were pooled and eluted into 20 fractions with an Ultremex SCX column containing 5 *μ*m particles (Phenomenex, USA). The eluted fractions were then desalted with a Strata X C18 column (Phenomenex, USA) and dried under vacuum [10].

### 2.4. Nano-HPLC-MS/MS

Each fraction was resuspended in buffer A (2% ACN, 0.1% FA) and centrifuged at 20,000*g* for 10 min. The final average concentration of peptide was about 0.5 *μ*g/*μ*l. 10 *μ*l supernatant was loaded on an LC-20AD nano-HPLC (Shimadzu, Kyoto, Japan) by the Autosampler onto a 2 cm C18 trap column. Then, the peptides were eluted into a 10 cm analytical C18 column (inner diameter of 75 *μ*m) packed in-house. The samples were loaded for 4 min at a speed of 8 *μ*L/min, and then they were run at 300 nL/min during the 44 min gradient. The gradient elution program was started from 2% to 35% B (98% ACN, 0.1% FA), followed by 2 min linear gradient to 80%, and maintained at 80% B for 4 min and finally returned to 5% in 1 min.

The peptides were subjected to nanoelectrospray ionization followed by tandem mass spectrometry (MS/MS) in a Q EXACTIVE (Thermo Fisher Scientific, San Jose, CA) coupled online to the HPLC. Intact peptides were detected in the Orbitrap with a resolution of 70000. Peptides were selected for MS/MS with high-energy collision dissociation (HCD) operating mode and the normalized collision energy was set at 27 V. The ion fragments were detected in the Orbitrap at a resolution of 17500. A data-dependent procedure that alternated between one MS scan and 15 MS/MS scans was applied for the 15 most abundant precursor ions. A threshold ion count of above 20000 was set in the MS survey scan with a following Dynamic Exclusion duration of 15 s. The applied electrospray voltage was 1.6 kV. Automatic gain control (AGC) was applied to optimize the spectra generated by the Orbitrap. The AGC target for full MS was 3*e*6 and 1*e*5 for MS2. For MS scans, the *m*/*z* scan range was 350 to 2000 Da. For MS2 scans, the *m*/*z* scan range was 100–1800 Da.

### 2.5. Database Screening

Raw data files acquired from the Orbitrap were converted into MGF files with Proteome Discoverer 1.2 (PD 1.2, Thermo). The MGF file was applied for search. Proteins identification was performed with Mascot search engine (Matrix Science, London, UK; version 2.3.02) against a database containing 143,397 sequences (*Homo sapiens*, release 2014_12).

For protein identification, a mass tolerance of 2 ppm was permitted for intact peptide masses and 0.05 Da for fragmented ions. One missed cleavage was allowed in the trypsin digests. The variable modifications were Gln- > pyro-Glu (N-term Q), oxidation (M), and deamidated (NQ); the fixed modifications were carbamidomethyl (C), iTRAQ 8-plex (N-term), and iTRAQ 8-plex (K). The charge state of peptides was set to +2 and +3. Specifically, an automatic decoy database search was performed in Mascot by choosing the decoy checkbox. A random sequence of databases was generated and tested for raw spectra as well as the real database. To reduce the probability of false peptide identification, only peptides (95% confidence interval) greater than “identity” in Mascot probability analysis were counted as identified. And at least one unique peptide was involved in each confidently identified protein.

Only protein with fold change meeting the criteria (>1.2 or <0.83) and *p* values < 0.05 was considered as protein with significant expression differences.

### 2.6. Function Method Description

Functional annotations of proteins were conducted with Blast2GO program against the nonredundant protein database (NR; NCBI). Metabolic pathways of these identified proteins were screened via KEGG database to filter proteins involved in any EH related pathway.

In addition to the above database search, on the other hand, candidate proteins were screened via searching in EH related literatures. Predicted protein-protein interactions were generated and visualized with STRING 10 software [11]. Clusters of proteins were determined through confidence level of surrounding protein-protein interactions.

According to the above analysis methods and literature retrieval, proteins closely related to the onset of EH were chosen as candidate special proteins.

### 2.7. ELISA and Western Blot

ELISA was employed to quantify the concentrations of the serum proteins, for determining the variation fold of expression level of candidate proteins. Human CTSG ELISA kit and human HYAL1 ELISA kit (Cusabio Biotech, Wuhan, Hubei, China), TGF-*β*1, and KNG1 (Abcam, Cambridge, MA, USA) were applied to determine the concentration of these proteins in each serum of validation set (27 HC and 27 EH individuals). Each sample was performed in duplicate according to ELISA manufacturer's instructions. KNG1 was the precursor of BK (bradykinin) in KKS pathway, which was a powerful endothelium-dependent vasodilator. The concentration of BK was also detected via ELISA kit (Cusabio Biotech, Wuhan, Hubei, China).

Protein KNG1 and CTSG were selected in verification with western blotting. A total of 50 *μ*g serum protein was separated in 12% w/v SDS-PAGE and transferred onto a PVDF membrane (0.22 *μ*m) with the semidry transfer system (Bio-Rad Trans-Blot Turbo, USA). After blocking with 5% skim milk in PBS buffer at room temperature for 2 h, the membrane was incubated with rabbit anti-human antibody (1 : 1000; Abcam, Cambridge, UK) at 4°C overnight. On the next day, membranes were incubated with HRP labeled goat anti-rabbit IgG (1 : 20000; ZSGB-BIO, China) at room temperature for 2 h after washing, followed by detection with ECL solution (Millipore, GER). The densitometry of bands was analyzed by Quantity One software (Bio-Rad, USA). Finally, membranes were blocked and incubated for internal marker development (rabbit anti-transferrin antibody, bs-2052R, Beijing, China) [12]. The gray value ratio of target proteins represented the relative expression levels between the two groups. The assay was repeated three times.

### 2.8. Statistical Analysis

All the measurement data was expressed as mean ± SD. For comparisons between two groups, all comparative data was analyzed with independent samples *t*-test or chi-square test. The SPSS16.0 statistical package was applied. A significance threshold of 0.05 was set and a threshold of *p* < 0.05 indicated a significant difference.

## 3. Results

### 3.1. Protein Identification and Candidate Proteins Screen

In the discovery phase, a total of 404 proteins were identified, with a false discovery rate of <1%. In the 404 proteins, the expression of 111 proteins was significantly differentiated between two groups (iTRAQ ratios ≥ 1.2 or ≤0.83), of which 81 proteins were downregulated and 30 proteins were upregulated. It also provided the information of protein ID, mass, sequence, and fold between two groups.

**Figure 2:**
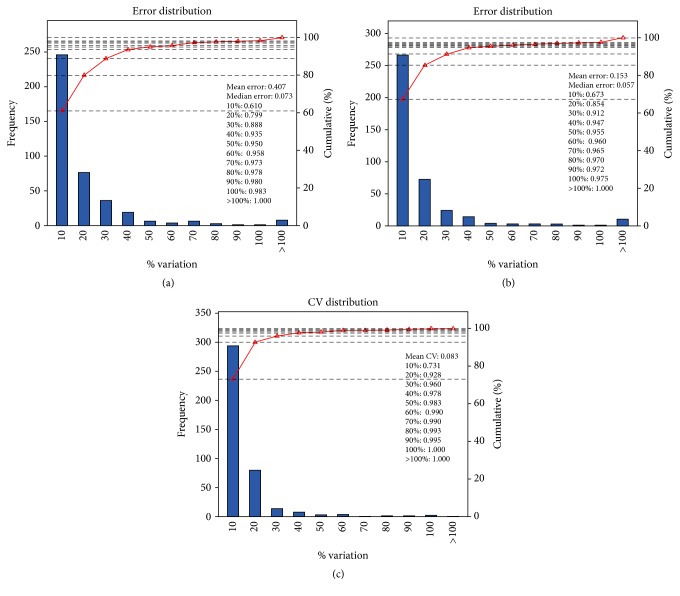
Reproducibility of proteomic analysis. The abscissa represents different variation levels; the left ordinate represents the number of quantitative proteins at different variation levels, and the right ordinate represents the accumulation ratio of total quantitative proteins at different variation levels. (a) and (b) display a comparison of the intragroup; for example, (a) shows that a difference less than variation of 0.1 could be observed in approximately 61.0% of the proteins (A1/A2) and a difference less than variation of 0.5 could be observed in more than 95.0% of the proteins. Besides, (c) exhibits the reproducibility of the comparison group. The mean CV of this comparison group was 8.3%, and when CV was 30%, the coverage ratio was up to 96% of all quantitative proteins between two comparison groups (A1 versus B1/A2 versus B2).

Reproducibility of the proteomic analysis was assessed to enhance the confidence of this study. To reduce individual or biodiversity difference, biological replicate was set in sample preparation. The subjects in each group were randomly divided into two subgroups. Four subgroups were formed: A1, A2, B1, and B2. The results of both intragroup replicate and repetitive assessment between comparison groups indicated a good repeatability (Figure 2).

**Figure 3:**
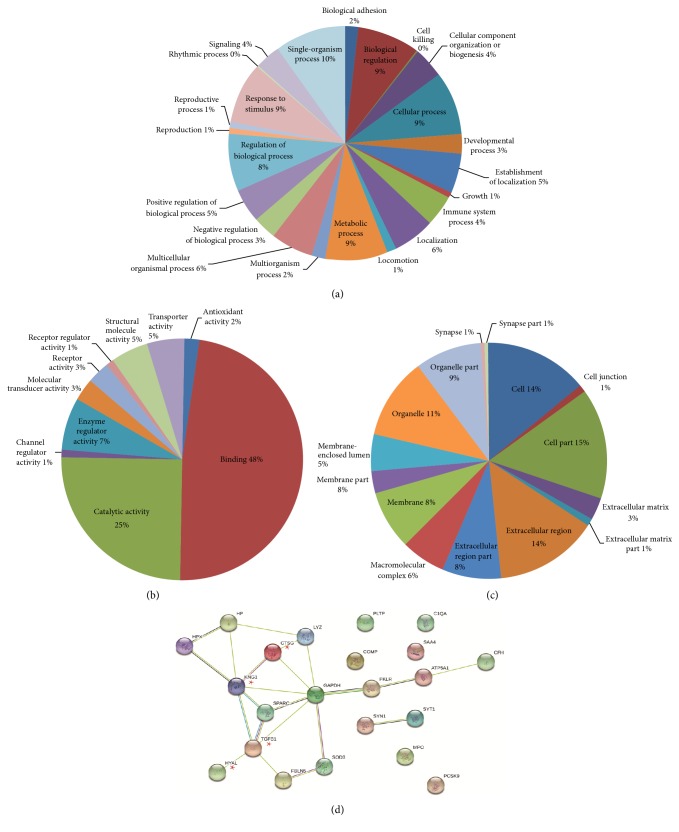
Bioinformatics analysis of differentially expressed proteins. GO analysis results showed biological process (a), molecular function (b), and cellular component (c). STRING analysis indicated visualization of protein-protein interactions for candidate special proteins in EH (d). In addition, 4 candidate special proteins verified by ELISA and western blot were marked with a red pentagram.

The associations of the 111 dysregulated proteins with onset and development of EH were explored. They were screened with GO, KEGG database, and literature review. Firstly, these proteins were searched in GO analysis and only 95 of the 111 dysregulated proteins were described in GO analysis. According to GO classification (Figure 3), it showed the biological process, molecular process, and cellular distribution of differentially expressed proteins, respectively. The dominant functions of these dysregulated proteins were provided (Figure 3(b)), including binding (48%), catalytic activity (25%), and enzyme regulator activity (7%). It implied that most dysregulated proteins acted as catalysts or regulators in biological pathways. The location of these proteins was also provided (Figure 3(c)). They were evenly located in the extracellular region (26%), cell and cell region (20%), organelle and organelle region (20%), and membrane (16%).

Target proteins would be diffused into blood, followed by having regulation effects in biological processes. These proteins were general secretory proteins, acting as link protein, enzyme, or regulator for biological functions. Thus, KEGG database and literatures were further searched. Combining with KEGG pathway and EH pathogenic mechanism associated literatures, 24 proteins out of 111 dysregulated proteins were screened out as candidates (the 24 candidate proteins with GO description are listed in Table 2). The genetic constitution or physiological or functional interaction of the 24 candidates was described with STRING network (Figure 3(d)). It displayed a close interaction among these proteins. In addition, to further investigate the plausible biological processes in which these proteins might be involved, we used bioinformatics analysis and the availability of commercial kits and discovered alterations in the biological process of factor production and catabolism process in many key pathogenesis nodes, and 4 candidates (proteins CTSG, KNG1, HYAL1, and TGF-*β*1) were involved in the process and were selected for further verification.

### 3.2. Validation of the Candidate Proteins

**Figure 4:**
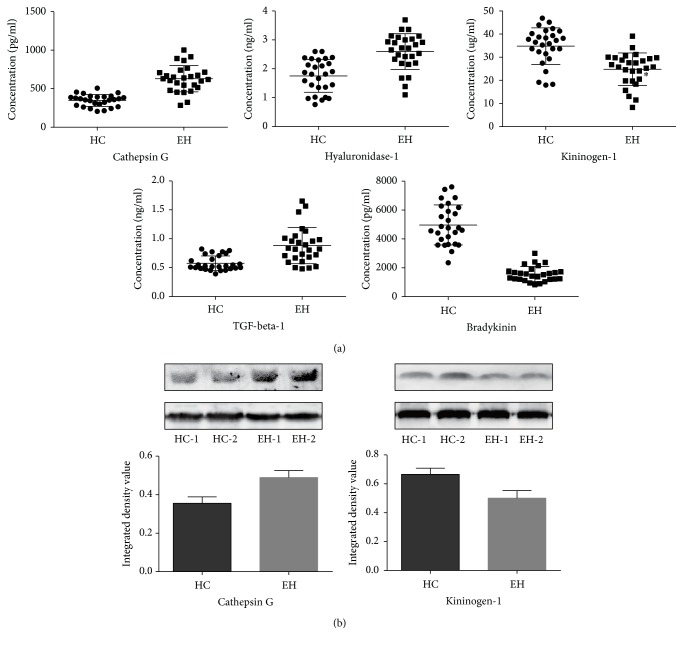
Verification of CTSG, HYAL1, KNG1, TGF-*β*1, and BK in serum. (a) The levels of these candidate special proteins and downstream substances were determined by ELISA in the serum of the HC group (*n* = 27) and EH group (*n* = 27). (b) Western blots of KNG1 and CTSG were performed. *p* values were calculated with independent samples *t*-test and all of the five proteins were significantly differentially expressed between the two groups (*p* = 0.000). Transferrin was applied as a loading control in western blot.

In this phase, 4 candidate special proteins were verified with an ELISA kit. 54 samples (27 HC individuals and 27 EH patients) participated in this verification and all standard curves were well fitted with *R*^2^ > 0.95 (Figure 4). The CTSG concentrations in serum of HC and EH groups were 347.58 ± 76.87 pg/mL and 629.22 ± 169.80 pg/mL. The levels of HYAL1 were 1.75 ± 0.57 ng/mL and 2.59 ± 0.62 ng/mL, respectively. The concentrations of KNG1 were 34.79 ± 7.89 ug/mL and 24.78 ± 7.06 ug/mL. The TGF-*β*1 levels were 0.58 ± 0.13 ng/mL and 0.88 ± 0.31 ng/mL in HC and EH groups, respectively. There was a significant difference in the concentration of all candidate proteins between the two groups (*p* < 0.01). In a word, the concentration profiles of the 4 proteins were consistent with the results in the discovery phase.

To confirm the differential expression observed in ELISA, the expression levels of CTSG (29 KD) and KNG1 (48 KD) were further examined with western blot. Transferrin (77 KD) was selected as an internal reference [13]. In Figure 4, the histogram of average gray ratio showed an increasing tendency of serum CTSG expression in EH group compared to that in HC group. However, it indicated a decreasing profile in serum KNG1. The western blot results of both candidates conformed to results of iTRAQ and ELISA.

### 3.3. Validation for the Downstream Substance of Candidate Special Proteins

RAAS and KKS were found as pathways in which the 4 proteins were involved. In the procedure of producing AngII and ALD, both CTSG and TGF-*β*1 participated in catalyzing or regulating this reaction as catalysis or key intermediate protein, while KNG1 is the precursor of BK. The levels of both AngII and ALD were obtained during patient recruitment. The results showed that both AngII and ALD were increased in individuals of EH group compared to HC group. This conformed to the results of special proteins. Besides, the level of BK was determined with ELISA kits. The average concentrations of BK in individuals of HC and EH groups were 4966.59 ± 1382.45 pg/mL and 1567.10 ± 520.26 pg/mL, respectively.

## 4. Discussion

EH is a worldwide disease with high incidence. BP was regulated by many biological materials, including genes, proteins, and metabolites. There was significant recent progress in studies on genomics and metabolomics, which revealed that the onset of EH was closely related to vascular endothelial and kidney dysfunction. However, the vital substances involved in these complicated pathological cases were not clearly demonstrated [14–16]. It is urgent to reveal which or what kind of substances had effects on raising BP.

Proteins are key components of biological networks with dynamic variations in different cells and organs during growth and development, in response to environmental stimuli, and in disease processes. The complex biological processes could be studied through systematic biology. With the help of computational tools, proteomic technologies were generally applied to demonstrate function activities from a system-wide perspective [17, 18].

It was aimed to explore the differentially expressed proteins between healthy individuals and EH patients. In this study, iTRAQ labeling coupled with LC-MS/MS analysis was applied to identify and quantify differentially expressed proteins in serum of EH patients. With these special proteins and their interaction, a biological network could be constructed, including discovered biological substances and pathological pathways. Various aspects of the pathological processes can be demonstrated and the mechanism can be further illustrated, providing new therapeutic targets for controlling EH.

Among differentially expressed proteins, 4 candidate proteins were considered to be the most close to EH and were further verified with ELISA and western blot. A similar profile of these proteins was observed in immunoassay verification and quantification proteomics. In a previous study, the 4 proteins have already been reported to be closely related to or to participate in pathophysiological processes of EH.

CTSG is secreted by the activated neutrophil, which is a serine protease, as well as a lysosomal enzyme [19]. It presents a similar activity to angiotensin converting enzyme (ACE), chymase, elastase, cathepsin B, and tonin during converting inactive decapeptide (AngI) to hypertensive peptide (AngII). In addition, CTSG is also capable of cleaving AngII directly from AGT (angiotensinogen). In normal conditions, all the above-mentioned physicochemical characteristics of CTSG assisted in the regulation of renin-angiotensin system (RAS) to maintain BP [20]. Protein TGF-*β*1 is also involved in the RAAS process. In previous publications, it was reported that BP elevation may be promoted by TGF-*β*1 through several mechanisms, such as stimulating endothelin mRNA expression in the vascular endothelium [21], suppressing renal tubular sodium reabsorption [22], and increasing renin release from juxtaglomerular cells in the kidney and AngII expression [23]. Besides, AngII also could induce time- and dose-dependent increases in TGF-*β* mRNA and activation, which directly results in excessive TGF-*β* [24]. Under hypertensive conditions, TGF-*β*1 expression would be increased by renin, AngII, and ALD. Thus, it can be seen that TGF-*β*1 and RAAS act synergistically to accentuate vasoconstriction and sodium and water retention during pathology progression of hypertension. From this, we can see that CTSG and TGF-*β*1 are both involved in the regulation of RAAS. Both proteins could stimulate AngII production, the essential bioactivator of RAS. If production of AngII exceeded the normal level, BP would be elevated through a variety of biologic activities including vasoconstriction [25], augmentation of venular permeability, and ALD production [26]. In our study, during both discovery and verification phases, there was a significantly higher expression of CTSG and TGF-*β*1 in EH group compared to that in HC group, and both proteins showed a positive correlation with AngII and ALD, respectively. The above results suggested that protein expression played an important role in the regulation of RAAS. In addition, we speculated whether proteins CTSG and TGF-*β*1 would become new hypertension treatment targets. In the future, inhibition of RAAS could be achieved also by reducing or blocking the generation and activation of the two proteins.

HYAL1 is the enzyme involved in the degradation of hyaluronan (HA). It is one of the most important subtypes in HYAL and is the predominant HYAL found in blood circulation [27, 28]. To our knowledge, the breakdown of HMW-HA to LMW-HA is associated with degradation of the endothelial glycocalyx and impairment of the vascular integrity [29]. HA loss would lead to various vascular abnormalities, such as attenuating NO content and activity, which would result in endothelial-dependent dilation dysfunction [30]. Karadag's investigation revealed that there was a significantly negative correlation between serum HYAL1 and NO level in serum of hypertension patients [31]. Similarly, our study found and validated that HYAL1 concentration was significantly higher in EH patients than in HC in both discovery and validation phases. We speculated that the generation of HYAL1 quantities sped up the degradation of HA, which indirectly led to NO reduction and arteriosclerosis in subjects with vascular endothelial dysfunction.

KNG1 is the essential factor of KKS and it could convert into high-molecular-weight kininogen (HMWK) and low-molecular-weight kininogen (LMWK), the precursors of BK [32]. It has commonly been confirmed that BK could induce production of NO, PGE2, and PGI2. As we know, both PGE_2_ and NO are the main endothelium-derived relaxing factors (EDRF) in humans [33–35]. So, to some extent, serum KNG1 level could affect the content of BK, PGE2, and NO to impact blood pressure. On the other hand, researchers from Kitasato University found that the KNG-deficient (BN-Ka) rats could hardly excrete sodium and water. These rats were extremely sensitive to ingested salt. Their research showed the deficiency of KNG or kinin production would directly led to the accumulation of sodium in the body followed by hypertension [16]. In our study, the expression of KNG1 was decreased in EH patients according to proteomics in discovery section and immunoassay in the verification phase. BK, as its product, was also validated via the ELISA kit. It was found that the level of BK was also decreased as the profile of KNG1, suggesting that there was a certain positive relationship between KNG1 and BK. So, any therapy increasing KNG1 production would improve the generation of BK and lower blood pressure.

**Figure 5:**
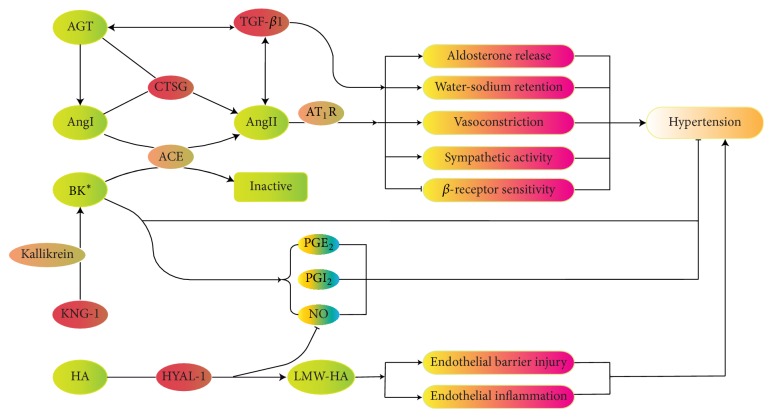
This map directly summarized the pathway of the 4 special proteins. All of these proteins were involved in RAAS and KKS. Among them, two proteins were enzymes, TGF-*β*1 was an intermediate protein, and KNG1 was a precursor of BK. BP could be affected by all these proteins through these pathways.

Our pilot study showed that the levels of a series of proteins, CTSG, TGF-*β*1, HYAL1, and KNG1, were significantly differentially expressed in EH patients. Their locations and functions in RAAS and KKS are displayed in Figure 5. From this map, it was observed that the 4 proteins played great roles in factor production and catabolism process in key pathogenesis nodes. So, this indicated that the pathophysiologic mechanism of EH may be mainly related to the level of enzymes and intermediate proteins, which has effects on the RAAS and KKS. Thus, we speculated that interfering in the concentration of these proteins, such as lowering the production or stimulating the degradation of HYAL1, CTSG, or TGF-*β*1, or aiding the production of KNG1 would be a new antihypertension strategy by influencing RAAS or KKS.

In this study, the involved key nodes were AngII, ALD, BK, and NO, all of which have been widely confirmed to be crucial components in BP regulation. Besides, exploring key special proteins could not only enhance understanding the pathological mechanism of EH systematically but also provide new therapeutic targets for the disease. Until now, the treatment and prognosis of hypertension have been limited in some certain patterns, without critical breakthrough. In this study, it was shown that BP could be influenced by the levels of target special proteins. In turn, BP would also be recovered after the level recovery of these proteins. So, these protein biomarkers could be potential therapeutic targets for EH in the future.

## 5. Conclusion

Biomarkers such as CTSG, TGF-*β*1, HYAL1, and KNG1 were jointly involved in RAAS and KKS, and their concentration level is integrated, consistent with BP variation. In addition, we noticed that enzymes or intermediate regulated proteins would be valuable factors in the occurrence and development processes of diseases. In the future, the 4 proteins would be further validated with larger samples. And we hope the discovery of these proteins may help pave the way for exploring new therapies of essential hypertension.

## Figures and Tables

**Table 1:** Clinical characteristics of the studied sample.

Clinical parameters	Healthy control	Essential hypertension	*p* value
Age (years)	54.98 ± 8.01	55.87 ± 7.41	0.599^a^
Gender (♂/♀)	26/21	25/22	0.500^b^
Smoker	13/47	16/47	0.012^b^
BMI	24.78 ± 2.40	24.63 ± 2.74	0.237^a^
Blood pressure (mmHg)			
Systolic	121.10 ± 10.80	150.38 ± 8.82	0.000^a^
Diastolic	74.66 ± 5.79	94.81 ± 10.93	0.000^a^
Angiotensin II (pg/ml)	52.52 ± 6.71	73.88 ± 7.72	0.000^a^
Aldosterone (pg/ml)	175.09 ± 33.70	218.86 ± 32.56	0.000^a^

*Notes*. Data was expressed as mean ± SD. ^a^*p* value between HC and EH individuals with independent samples *t*-test. ^b^Chi-square test; *p* ≤ 0.05 was considered to be statistically significant. BMI = weight (kg)/height^2^ (m^2^).

**Table 2:** Molecular functions, biological processes, and expression levels of 24 differentially expressed proteins.

Protein ID	Protein name	Molecular function	Biological process	A/B
sp|P08311	Cathepsin G	Binding Catalytic activity	Proteolysis Regulation of immune response	0.7645

tr|G3XAP6	Cartilage oligomeric matrix protein	Binding Structural molecule activity	Cell adhesion	1.265

tr|Q0QET7	Glyceraldehyde-3-phosphate dehydrogenase	Binding Catalytic activity	Oxidation-reduction process	0.842

sp|P05164-2	Myeloperoxidase	Antioxidant activity Binding Catalytic activity	Hydrogen peroxide catabolic process Oxidation-reduction process	0.6755

sp|P01137	Transforming growth factor beta-1	Binding	Epidermal growth factor receptor signaling pathway	0.7945

tr|B3KUI5	Hyaluronidase	Binding Catalytic activity Receptor activity	Hyaluronan biosynthetic process Positive regulation of hyaluronan cable assembly Response to reactive oxygen species	0.8355

tr|D3DQH8	Secreted protein	Binding	Response to glucocorticoid stimulus Response to calcium ion	0.7905

tr|A8MUB1	Tubulin alpha-4A	Binding Catalytic activity Structural molecule activity	Protein polymerization Platelet degranulation Platelet activation	0.8095

tr|J3KQA0	Synaptotagmin I	Binding Transporter activity	Detection of calcium ion Glutamate secretion	0.7595

sp|P61626	Lysozyme C	Catalytic activity	Inflammatory	1.213

tr|D3DNU8	Kininogen-1	Binding Enzyme regulator activity	Elevation of cytosolic calcium ion Positive regulation of renal sodium excretion; smooth muscle contraction; vasodilation; inflammatory response	1.2835

sp|P02790	Hemopexin	Binding Transporter activity	Cellular iron ion homeostasis; positive regulation of humoral immune response mediated by circulating immunoglobulin	1.864

sp|P25705-2	ATP synthase subunit alpha	Binding Catalytic activity Transporter activity	ADP biosynthetic process ATP catabolism Negative regulation of endothelial cell proliferation	0.847

sp|P17600-2	Synapsin-1	Binding Catalytic activity Transporter activity	Neurotransmitter secretion	0.7225

tr|M0R116	Sodium/potassium-transporting ATPase subunit	Binding Catalytic activity Transporter activity	ATP biosynthetic process Transmembrane transport	0.807

sp|Q8NBP7	Proprotein convertase subtilisin/kexin type 9	Binding Catalytic activity Channel regulator activity	Proteolysis Negative regulation of catalytic activity Negative regulation of receptor recycling	0.8745

tr|B4DNK4	Pyruvate kinase	Binding Catalytic activity	Small molecule metabolic process Glycolysis	0.816

tr|H0YJ31	Fibulin-5	Binding Molecular transducer activity Receptor activity	Regulation of removal of superoxide radicals	0.774

sp|P00738	Haptoglobin	Binding Catalytic activity	Negative regulation of hydrogen peroxide catabolic process; proteolysis; negative regulation of oxidoreductase activity	1.3475

sp|P08603	Complement factor H	—	Regulation of complement activation	0.7905

tr|B3KUE5	Phospholipid transfer protein	Binding	Vitamin E biosynthetic process	0.7085

sp|P08294	Extracellular superoxide dismutase	Antioxidant activity Binding Catalytic activity	Removal of superoxide radicals	0.8715

sp|P02745|	Complement C1q subcomponent subunit A	Binding	Cell-cell signaling Complement activation, classical pathway	1.5315

tr|B2R5G8|	Serum amyloid A protein	—	Acute-phase response	0.603
